# A novel scoring system for the early detection of anastomotic leakage: bedside leak score—a pilot study

**DOI:** 10.3389/fsurg.2023.1204785

**Published:** 2023-07-28

**Authors:** Ibrahim H. Ozata, Emre Bozkurt, Serkan Sucu, Salih N. Karahan, Furkan Camci, Feyza Cetin, Emre Ozoran, Orhan Agcaoglu, Emre Balik, Dursun Bugra

**Affiliations:** ^1^ Department of General Surgery, Koç University School of Medicine, Istanbul, Turkey; ^2^ Koc University School of Medicine, Istanbul, Turkey; ^3^ Department of General Surgery, VKV American Hospital, Istanbul, Turkey

**Keywords:** colorectal cancer, colon cancer, colorectal surgery, anastomosis, anastomotic leakage, scoring systems

## Abstract

**Background:**

Anastomotic leakage is a major complication in colorectal surgery, resulting in significant morbidity and mortality rates. Despite substantial progress in surgical technique, anastomotic leakage rates remain stable. An early diagnosis of anastomotic leaks was proven to reduce adverse outcomes and improve survival.

**Objective:**

This study aims to find a novel scoring system for detecting anastomotic leaks using inflammatory and nutritional indicators after colorectal surgery. Our purpose was to analyze the diagnostic accuracy of leak scores $\left(\frac{\left(\mathrm{CRP}\;\mathrm{POD}3\right)}{(\mathrm{CRP}\;\mathrm{POD}1)*\mathrm{preoperative}\;\mathrm{albumin}\;\mathrm{level}}\right)$ in predicting postoperative complications.

**Design:**

The study included colorectal cancer patients who underwent curative surgery at Koc University Hospital between 2014 and 2018. Patients were categorized into two groups depending on the presence of anastomotic leaks and compared in terms of preoperative albumin levels, CRP levels in postoperative days 1 and 3, anastomotic leakage rates, length of hospital stay, and CRP quotient, which was calculated by dividing POD 3 CRP level to POD 1 CRP level. The bedside leak score is calculated by dividing the CRP quotient by the preoperative albumin level. The predictive value of bedside leak score, CRP quotient, and preoperative albumin levels in estimating anastomotic leakage was analyzed, and a cutoff value for the leak score was calculated.

**Results:**

A total of 184 patients were included in the study. The leak score, CRP POD 3–1 ratio, and preoperative albumin levels were found to successfully detect anastomotic leakage. The area under the curve for the leak score was calculated as 0.78. The optimal cutoff value was found to be 50.3 for the bedside leak score, which shows 90.9% sensitivity and 59.3% specificity.

**Conclusion:**

The leak score may represent a valuable diagnostic tool for detecting patients at risk for anastomotic leakage after colorectal surgery and planning a better strategy to reduce morbidity and mortality rates and associated costs. However, further multicenter studies with large cohorts are necessary to confirm these results.

## Introduction

Anastomotic leakage (AL) is considered the “Achilles heel” of gastrointestinal tract surgery and is a devastating complication of colorectal surgery. Even in very experienced colorectal surgery centers, the incidence rate of AL is approximately 7% (1, 2). Anastomotic leakage can cause significant morbidity and mortality rates, resulting in decreased disease-free and overall survival along with higher healthcare costs (3–5). Despite recent advances in surgical techniques and perioperative care, the incidence rate of AL remains unchanged (6). Nonetheless, an early diagnosis of AL can prevent adverse short- and long-term outcomes by reducing the rate of permanent stomas and cancer recurrence, which leads to prolonged long-term survival (7).

Once an AL becomes clinically overt, it is usually too late to prevent complications and adverse clinical outcomes. Therefore, risk assessment and prediction scores for AL are crucial in colorectal surgery (8–14). C-reactive protein (CRP), a commonly used inflammatory marker in patient care after colorectal surgery, has clinical significance for detecting infectious complications. However, the CRP level alone does not reflect a patient's nutritional status, including hypoalbuminemia, which is a well-known risk factor in detecting AL (15–17). The CRP/albumin ratio (CAR) was found to have higher predictivity and accuracy. However, the CAR lacks clarity in the trajectory of CRP, which was shown to help rule out AL (11). C-reactive protein reaches its peak value 3 days after surgery, but increased values are not always associated with complications. As a result, a new score that incorporates the change in the inflammatory state and the nutritional status which can be practically calculated at the bedside is needed (11, 13, 18).

This study aims to find a novel scoring system for detecting colorectal AL using inflammatory and nutritional indicators. The CRP quotient on postoperative days 1 and 3 (POD1 and POD3) is divided into preoperative albumin levels and multiplied by 100 to obtain a new score: the bedside leak score. This study's purpose is to establish the success of the bedside leak score in detecting AL in advance.

## Materials and methods

**Figure 1:**
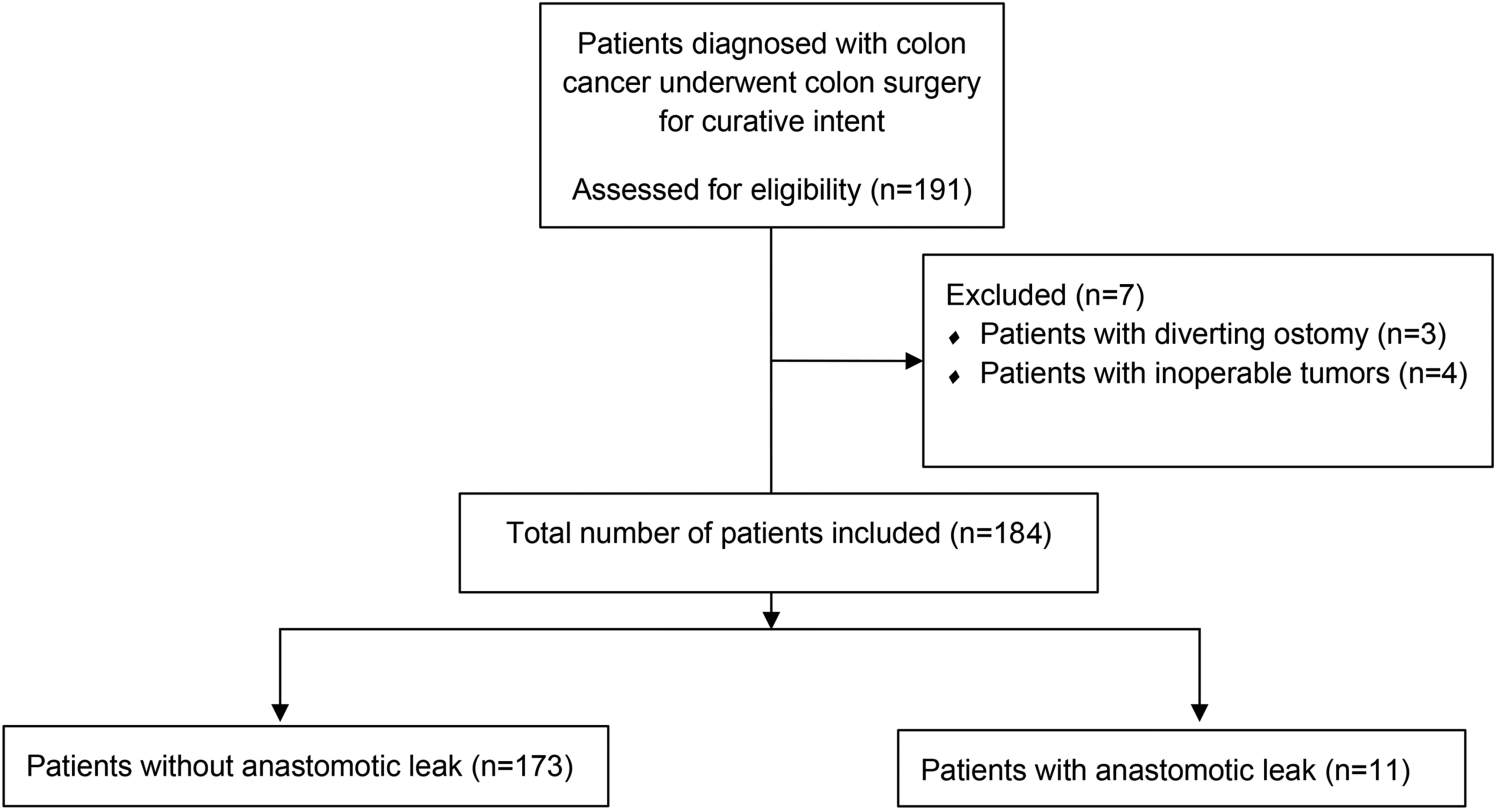
CONSORT diagram.

This study included patients with a histopathological diagnosis of colon cancer who underwent curative surgery between 2014 and 2018 in a tertiary care colorectal surgery clinic. Patient records were prospectively collected and retrospectively analyzed. Data, including patient demographics, tumor location, type of tumor pathology, Clavien–Dindo score, mortality within 30 days, surgical procedure, type of surgery, preoperative albumin levels, CRP levels on POD 1 and 3, CAR on POD 1 and 3, time of operation, day of diagnosis of AL, presence of AL, and length of hospital stay, were obtained (Figure 1). The study excluded patients who have inoperable tumors, underwent emergent or urgent surgery, received neoadjuvant treatment, and have diverting ostomies. We did not routinely create a diverting ostomy unless the patient had a tumor that caused an obstruction. A diagnosis of AL was based on clinical suspicion confirmed by extraluminal contrast extravasation, gas-containing collections around anastomosis shown on computed tomography scans with oral contrast, or anastomotic disruption seen on endoscopy.

**Figure 2:**
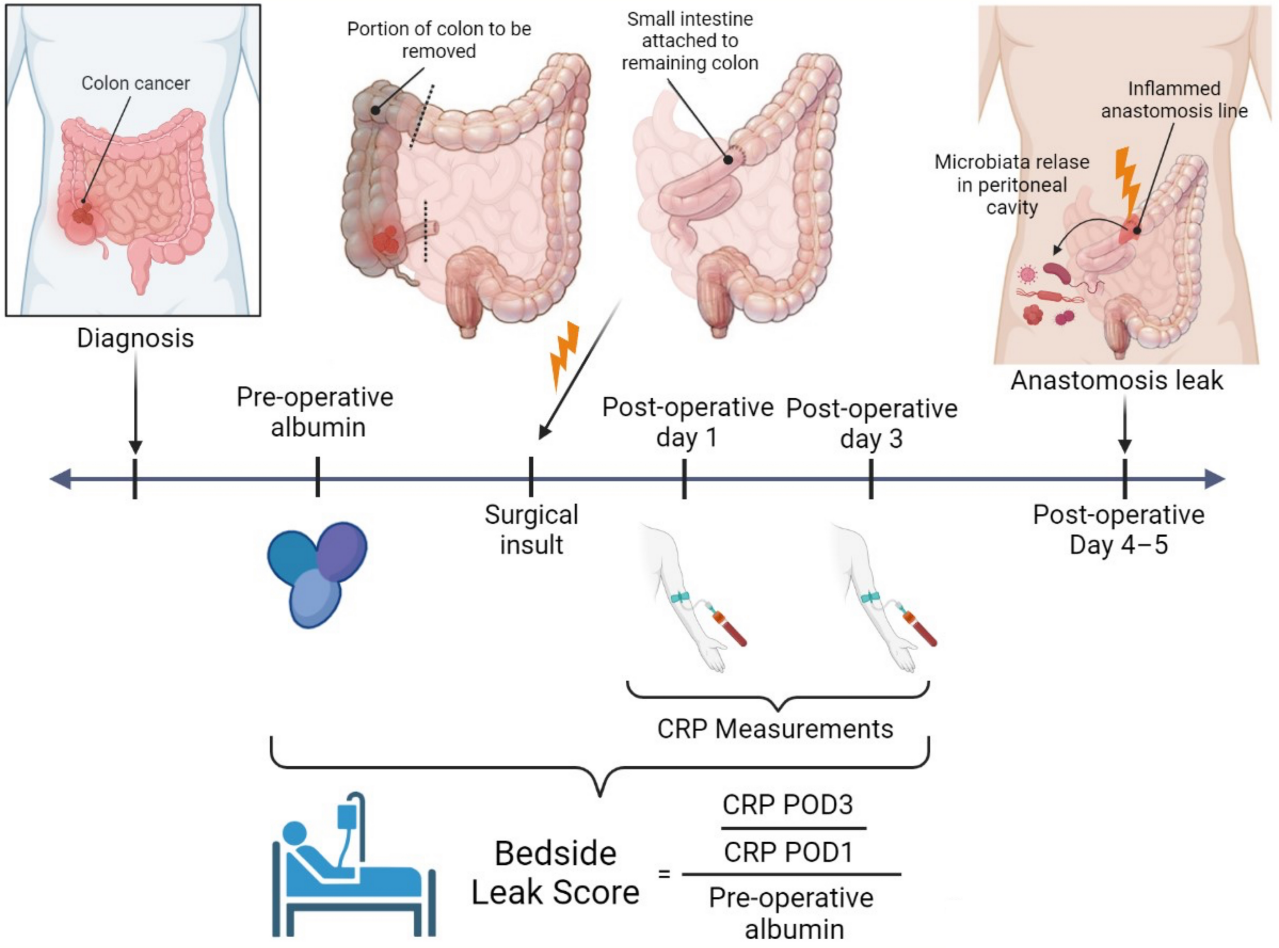
Bedside leak score—created with BioRender.com.

The CRP quotient on POD 1 and 3 was divided into preoperative albumin levels and multiplied by 100 to obtain the bedside leak score for all patients (Figure 2).

This study was approved by the Koc University Ethics Committee with Approval No. 2021. 314.IRB1.140, and all patients filled out a written informed consent form for participation and consent for publication. The study was conducted in accordance with the principles of the Declaration of Helsinki.

Statistical analysis was performed using SPSS version 26 software (IBM, USA). The bedside leak score, POD 1 and POD3/POD1 (dynamic) CRP levels, CAR on POD1 and POD3, and albumin levels in patients who experienced AL were compared with those of the patients who did not have AL using the independent samples Kruskal–Wallis test. The patient's age, body mass index (BMI), and CRP levels on POD3 were tested using an independent sample *T*-test. The Mann–Whitney *U*-test was used to compare the length of hospital stay and operative time in patients with and without AL. Receiver operating characteristic (ROC) curves were obtained for the dynamic CRP levels, CRP on POD1 and POD3, CAR on POD1 and POD3, and bedside leak score to estimate AL. The area under the ROC curve values was divided into four groups: >0.90 = excellent, 0.80–0.90 = good, 0.70–0.80 = fair, and <0.60 = poor. A statistically significant value was accepted at *p* ≤ 0.050.

## Results

After excluding patients with inoperable tumors (*n* = 4) and diverting ostomies (*n* = 3), this study included 183 patients. Table 1 shows the clinical data of the patients. The two groups were comparable in terms of patient demographics (gender, age, BMI, and ASA scores). The Clavien–Dindo scores were found to be higher in the AL group. No mortality was observed in the AL group. The length of hospital stay was significantly longer in patients with AL (23.5 vs. 8.7 days, *p *< 0.010). The operative time was found to be longer in the AL group (209 vs. 180 min), despite failing to reach statistical significance.

**Table 1:** Patient demographics, perioperative outcomes, and histopathological analysis.

Patient demographics	Anastomotic leak			*p*-Value
	Absent (*n* = 173)	Present (*n* = 11)	Total (*n* = 184)	
Gender				
Male (%)	97 (56.1)	9 (81.8)	106 (57.6)	0.094
Female (%)	76 (43.9)	2 (18.2)	78 (42.4)	
Age (±SD)	62.7 ± 14.6	64.7 ± 16.9	62.8 ± 14.7	0.708
BMI (±SD)	26.4 ± 4.0	25.6 ± 3.4	26.3 ± 3.9	0.758
ASA score (IQR)	2 (1–3)	2 (1–3)	2 (1–3)	0.571
Tumor location				
Ascending (%)	58 (33.5)	3 (27.3)	61 (33.2)	0.671
Transverse (%)	22 (12.7)	3 (27.3)	25 (13.6)	
Descending (%)	17 (9.8)	0	17 (9.2)	
Sigmoid (%)	56 (32.4)	4 (36.4)	60 (32.6)	
Rectosigmoid (%)	15 (8.7)	1 (9.4)	16 (8.7)	
Synchronous (%)	5 (2.9)	0	5 (2.7)	
Resection type				
Right hemicolectomy (%)	68 (39.3)	3 (27.3)	71 (38.6)	0.750
Left hemicolectomy (%)	9 (5.2)	0	9 (4.9)	
Subtotal colectomy (%)	22 (12.7)	2 (18.2)	24 (13.0)	
Total colectomy (%)	7 (4)	1 (9.1)	8 (4.3)	
Anterior resection (%)	67 (38.7)	5 (45.5)	72 (39.1)	
Type of surgery				
Open (%)	26 (15)	2 (18.2)	28 (15.2)	0.660
Laparoscopic (%)	125 (77.2)	9 (81.8)	134 (72.8)	
Robotic (%)	13 (7.5)	0	13 (7.1)	
Type of pathology				
Adenocarcinoma (%)	121 (70.8)	6 (54.5)	127 (69.8)	0.479
Mucinous adenocarcinoma (%)	46 (26.9)	5 (45.5)	51 (28)	
Signet ring cell (%)	4 (5)	0	4 (2.2)	
Clavien–Dindo score				
1	97 (56.1)	0	97 (52.7)	**<0** **.** **001**
2	47 (27.2)	1 (9.1)	48 (26.1)	
3	24 (13.9)	8 (72.7)	32 (17.4)	
4	3 (1.7)	2 (18.2)	5 (2.7)	
5	2 (1.2)	0	2 (1.1)	
Operative and postoperative features				
Leakage on POD (IQR)	0	6 (5–8)	N/A	N/A
Mortality within 30 days (%)	3 (1.6)	0	3 (1.6)	0.660
Length of hospital stay (±SD)	8.68 ± 6.51	23.5 ± 8.5	9.5 ± 7.5	**<0** **.** **001**
Operative time (±SD)	180.6 ± 116.7	209 ± 116.6	182.4 ± 116.5	0.212

SD, standard deviation; ASA, American Society of Anaesthesiologists' classification of Physical Health; BMI, body mass index; IQR, interquartile range; POD, postoperative day.

Bold values represent statistically significant results (*p*<0.05).

**Figure 3:**
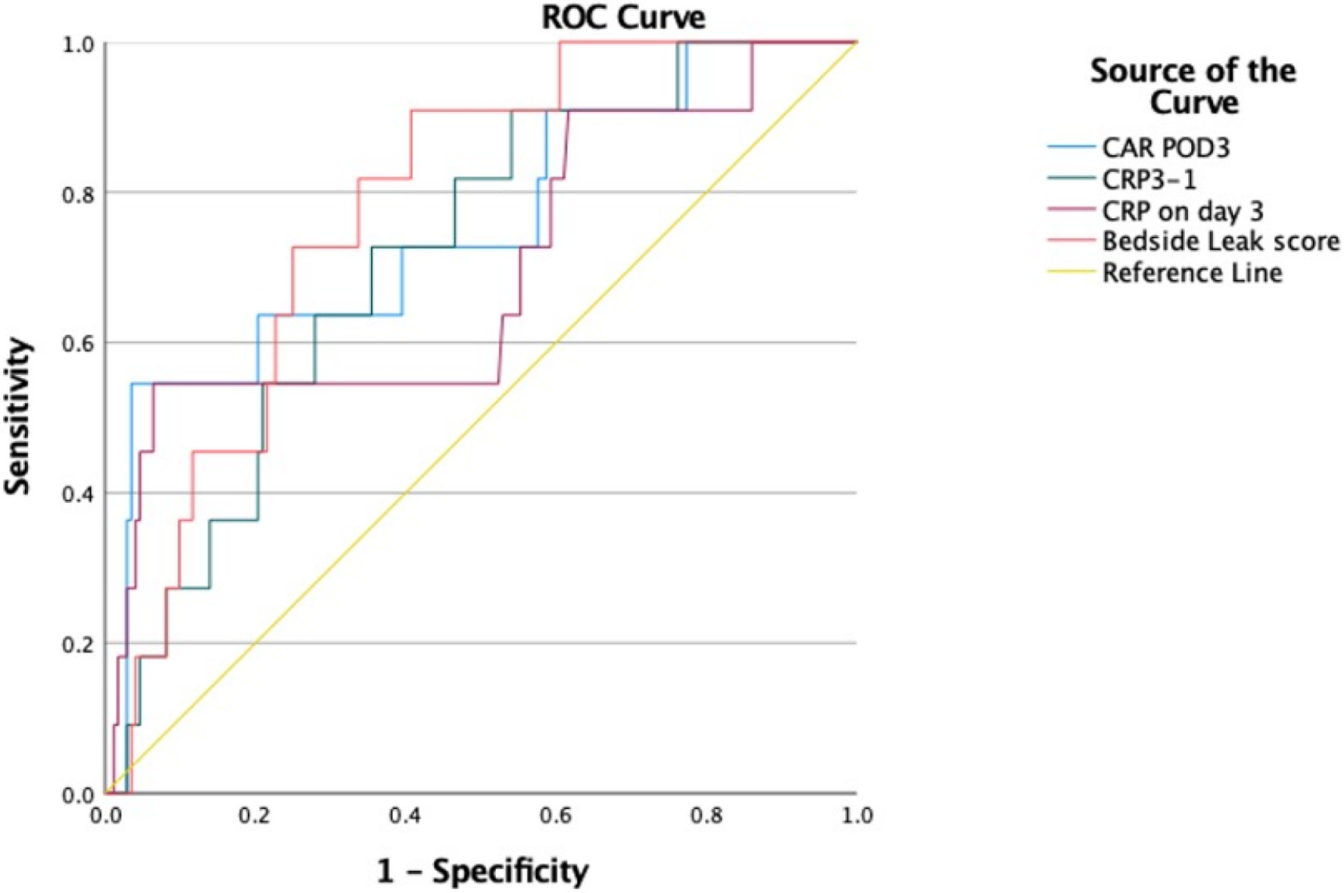
ROC curves for bedside leak score, CRP 3–1 ratio, CRP on day 3, and CRP to albumin ratio in postoperative day 3.

The association between AL and preoperative albumin levels, bedside leak score, CAR on POD3, and dynamic CRP (CRP POD3/POD1) was statistically significant with *p*-values of 0.001, 0.002, 0.005, and 0.016, respectively (Table 3). Furthermore, to compare diagnostic values among bedside leak score, dynamic CRP levels, CRP on POD1 and POD3, and CAR on POD1 and POD3, ROC curves were generated, and the areas under the curve (AUCs) for each diagnostic parameter were calculated (Tables 2, 3). The AUCs for the bedside leak score, CRP POD 3–1 ratio, CRP POD3, and CAR POD3 were 0.781, 0.717, 0.695, and 0.753, respectively (Figure 3 and Table 2). The bedside leak score was found to have a sensitivity of 90.9, with a cutoff value of 50.7, which shows the lowest false positive ratio among them (Tables 4, 5).

**Table 2:** Diagnostic values of different models.

Variable	Area under the curve	*p*-Value	Lower and upper bound
CRP on POD1	0.425	0.404	0.294–0.556
CRP on POD3	0.695	0.070	0.510–0.880
CRP on POD3/POD1	0.717	**0** **.** **016**	0.580–0.854
CAR on POD1	0.506	0.944	0.359–0.654
CAR on POD3	0.753	**0** **.** **005**	0.589–0.916
Bedside leak score	0.781	**0** **.** **002**	0.672–0.889

CRP, C-reactive protein; POD1, postoperative day 1; POD3, postoperative day 3; CAR, C-reactive protein/albumin ratio.

**Table 3:** Calculation of bedside leak score.

Parameters	Patient	Preoperative	POD1	POD3	POD1/POD3 (dynamic CRP)	(POD1/POD3)/albumin (bedside leak score)
Albumin	AL	3.59 ± 0.62	-	-	-	-
	Non-AL	4.14 ± 0.45	-	-	-	-
	*p*-Value	**0.001**	-	-	-	-
CRP	AL	-	53.7 ± 24.5	206.2 ± 102	4.5 ± 3.25	124.5 ± 86.2
	Non-AL	-	73.5 ± 57.7	138.5 ± 82.0	2.93 ± 3.09	71.6 ± 99.7
	*p*-Value	-	0.404	0.070	**0.016**	**0.002**
CAR	AL	-	15.6 ± 7.73	57.6 ± 26.9	-	-
	Non-AL	-	18.4 ± 16.2	34.1 ± 21.8	-	-
	*p*-Value	-	0.944	**0.005**	-	-

POD1, postoperative day 1; POD3, postoperative day 3; AL, anastomotic leakage; CRP, C-reactive protein; CAR, C-reactive protein/albumin ratio.

**Table 4:** Statistical properties of different predictive scores.

Parameters	Cutoff value	Sensitivity (%)	Specificity (%)	PPV	NPV
CRP on POD3	105.3	90.9	39	0.086	0.985
Dynamic CRP	1.75	90.9	45.9	0.096	0.987
CAR on POD3	26.22	90.9	41.3	0.098	0.986
Bedside leak score	50.7	90.9	59.3	0.125	0.990

**Table 5:** Predictive value of bedside leak score.

Parameters	AL+	AL−	Predictive value
Bedside leak score >50	10	70	0.125 (+)
Bedside leak score <50	1	103	0.990 (−)
Sens and spec	90.9	59.3	

POD3, postoperative day 3; AL, anastomotic leakage; CRP, C-reactive protein; CAR, C-reactive protein/albumin ratio; PPV, positive predictive value; NPV, negative predictive value.

## Discussion

Compared with other traditional diagnostic measures, including preoperative albumin levels and CRP levels on postoperative days 1 and 3, the bedside leak score provides statistically valuable diagnostic predictive power. With a cutoff value of 50.7, the sensitivity of the bedside leak score is 90.9, which is a significant value for a screening score.

Anastomotic leakage is one of the most devastating complications of colorectal surgery because of its high morbidity and mortality rates. Early AL prediction and diagnosis AL improve short-term morbidity and mortality rates and long-term survival (7). Therefore, substantial efforts are made to predict and prevent this complication, especially after colon cancer surgery (19, 20).

To detect AL early and prevent morbidity, several blood markers and extravascular fluid biomarkers, including CRP levels, are used in many studies. In addition, the clinical use of the spot and trajectory values of CRP (11, 13), CAR (18, 21), and novel systemic inflammatory biomarkers (10) is still under investigation in several surgical practices.

This study examined the accuracy of dynamic CRP changes (POD3 to POD1), spot CRP values (POD1 and POD3), CAR on POD 1 and 3, and the bedside leak score (dynamic CRP/albumin) in predicting AL. The results indicated that the bedside leak score was the most accurate predictive test.

Looking at the predictive value of CRP, one of the key differences between this study and previous ones is that this study assessed dynamic CRP rather than spot values (13). The inflammatory response due to AL increases gradually. Therefore, systemic inflammatory markers such as CRP increase progressively in response to AL. A postoperative increase in CRP is expected, but the level depends on the degree of surgical damage. Using daily spot values of CRP to assess evolving conditions is less likely to be accurate. A test to evaluate dynamic changes is more advantageous than an isolated spot value because AL gradually develops (11). This study also reveals how the CRP levels of patients with AL exponentially increased compared with those of the non-AL group, highlighting the importance of the trajectory model.

This study evaluated the prediction of AL with a dynamic CRP to preoperative albumin ratio (the bedside leak score), which is the trajectory model. The CAR is the arbitrary CRP to albumin ratio (18, 21). In this study, albumin is preferred because it is a good predictor of malnutrition and has been proven to be an early detection marker for postoperative complications. Low preoperative albumin levels increase the risk of postoperative complications such as AL (15, 22). Previous studies used the CAR to determine the prognosis and overall survival of cancer patients (23–25). On the other hand, only a few articles use CAR to predict AL following colorectal cancer surgery (18, 21), and only one article used the trajectory model of CRP to predict AL (11). However, no studies in the literature specifically anticipated AL by considering the ratio of dynamic CRP changes to preoperative albumin levels.

Few articles investigated the predictive value of inflammatory biomarkers [interleukin (IL)-1β, IL-6, IL-10, TNF-α, and MMP-2 and -9] in the peritoneal fluid of cancer patients who underwent surgery and experienced AL in different locations (11, 26, 27). Even though peritoneal fluid biomarkers have a higher potential of detecting AL than systemic markers, Shi et al. (26) stated that investigating such inflammatory markers in the peritoneal fluid cannot be applied in every institution due to their low cost-effectivity and limited availability of kits. The components of the bedside leak score (i.e., the preoperative albumin and the CRP on POD1 and POD3) are commonly used blood parameters in patient care after colon cancer surgeries. Therefore, the bedside leak score prevents additional costs and provides a tremendous economic advantage by avoiding complications due to AL.

The high sensitivity score and AUC of the bedside leak score make this test highly valuable for AL screening. Smith et al. (28) showed high sensitivity of the trajectory model of CRP values. However, this model has low specificity compared with the bedside leak score. This finding emphasizes that the bedside leak score is more accurate in predicting AL, with lower false positive results, than the trajectory model. Therefore, using the bedside leak score may prevent unnecessary tests or interventions aimed at confirming the diagnosis of AL. Paliogiannis et al. (18) revealed that the CAR had great potential in predicting AL with very low false positive rates. However, compared with the bedside leak score, its sensitivity is lower as a screening test for AL. The high sensitivity and relatively high specificity of the bedside leak score are crucial for anticipating AL and preventing unnecessary, costly diagnostic tests.

The bedside leak score also adds objectivity to clinical suspicion. In clinical practice, evaluating patients for potential AL includes a physical examination, drain inspection, and close monitoring of postoperative vital signs. Fever, abdominal pain, nausea and vomiting, low blood pressure, and tachycardia are some clinical symptoms of AL. Hence, suspicion of an AL is up to the surgeon's clinical experience. The subjectivity of the assessment may cause delays in an interventional attempt to prevent complications from AL, especially in centers with low experience. However, the bedside leak score can predict AL before any symptoms appear. In a study that was validated prospectively, an online objective anastomotic risk calculator was found to be a remarkably reliable tool in predicting AL. Differences in risk perception, personal risk tolerance, or the pressure of external biases could affect surgeons’ decisions before taking action (29). Surgeons’ personal experience can also affect their assessment. For these reasons, it is vital to have a scoring system based on patient data, allowing the surgeon to evaluate the situation objectively. A subjective scoring system such as the bedside leak score allows other healthcare providers to verify the condition with the same impartiality.

Fluorescent-guided surgery, particularly the use of indocyanine green, has emerged as a valuable tool for the prediction of anastomotic leakage in colorectal surgery. However, indocyanine green was not routinely used in this cohort of patients as the study enrollment period ended in 2018. However, in recent years, with promising reports on the use of intraoperative fluorescence imaging in evaluating anastomosis perfusion, we have started to incorporate this technique into our routine clinical practice, especially during minimally invasive surgeries (30, 31). The addition of ICG to the bedside leak score might be valuable and can increase its predictive value. Future studies might consider utilizing ICG in scoring systems.

There were several limitations to this study. First, this investigation was performed in a single center, and the sample size of this retrospective study was not big enough to validate the bedside leak score internally and externally. As surgical technique and perioperative management are crucial in postoperative complications, multicentric and prospective studies are necessary to confirm the results of this study. In addition, one of the major limitations of this study is that it is a retrospective study and relies on previously collected data. The study's retrospective nature makes it susceptible to selection and information bias.

## Conclusion

This retrospective study compared the novel bedside leak score with commonly used serum biomarkers and newly used formulations in predicting AL following colon cancer surgeries. Among them, the bedside leak score, with a cutoff value of 50, was the most accurate in terms of providing preoperative risk evaluation and predicting AL based on the trajectory of CRP levels. Its high sensitivity indicates that the bedside leak score may represent a promising screening tool for predicting AL and assist surgeons in planning a better strategy to reduce morbidity and mortality rates and associated healthcare costs. However, due to the inherent limitations of the study design, further multicenter studies with large cohorts are necessary to confirm the real value of this score in clinical practice.

## Data Availability

The raw data supporting the conclusions of this article will be made available by the authors, without undue reservation.
